# Effect of novobiocin on the viability of human gingival fibroblasts (HGF-1)

**DOI:** 10.1186/2050-6511-15-25

**Published:** 2014-05-01

**Authors:** Anna K Szkaradkiewicz, Tomasz M Karpiński, Andrzej Szkaradkiewicz

**Affiliations:** 1 Department of Conservative Dentistry and Periodontology, University of Medical Sciences, Bukowska 70, str., 60-812, Poznań, Poland; 2 Department of Medical Microbiology, University of Medical Sciences, Wieniawskiego 3, str., 61-712 Poznań, Poland

**Keywords:** Novobiocin, Hsp90, Human gingival fibroblasts, Viability, Cell death

## Abstract

**Background:**

Novobiocin is a coumarin antibiotic, which affects also eukaryotic cells inhibiting activity of Heat shock protein 90 (Hsp90). The Hsp90 represents a molecular chaperone critical for stabilization and activation of many proteins, particularly oncoproteins that drive cancer progression. Currently, Hsp90 inhibitors focus a significant attention since they form a potentially new class of drugs in therapy of cancer. However, in the process of tumorigenesis a significant role is played also by the microenvironment of the tumour, and, in particular, by cancer-associated fibroblasts (CAFs). This study aimed at examination of the effect played by novobiocin on viability of human gingival fibroblasts (HGF-1).

**Methods:**

The studies were conducted using 24 h cultures of human gingival fibroblasts – HGF-1 (CRL-2014) in Chamber Slides, in presence of 0.1, 0.5, 1.0, 2.5 or 5.0 mM novobiocin. Cell viability was evaluated using fluorescence test, ATP assay and LDH release.

**Results:**

Viability of HGF-1 was drastically reduced after 5 hour treatment with novobiocin in concentrations of 1 mM or higher. In turn, the percentage of LDH-releasing cells after 5 h did not differ from control value although it significantly increased after 10 h incubation with 1 mM and continued to increase till the 20th hour.

**Conclusions:**

The obtained data indicate that novobiocin may induce death of human gingival fibroblasts. Therefore, application of the Hsp90 inhibitor in neoplastic therapy seems controversial: on one hand novobiocin reduces tumour-associated CAFs but, on the other, it may induce a significant destruction of periodontium.

## Introduction

Novobiocin represents a coumarin antibiotic produced by *Streptomyces spheroides* and *Streptomyces niveus* strains and manifesting activity against Gram-positive bacteria [1]. The antibiotic exerts mainly bacteriostatic activity, inhibiting function of ATP-dependent gyrase [2,3]. In addition, in recent years novobiocin was found to act also on eukaryotic cells, blocking chaperone activity of 90 kDa heat shock proteins (Hsp90) through competitive binding to the Hsp90 C-terminal ATP binding site [4,5]. Due to inhibition of Hsp90, many oncoproteins linked to all six hallmarks of cancer progression (angiogenesis, immortalization, metastasis, impaired apoptosis, insensitivity to antigrowth signals and autocrine growth) undergo degradation in cancer cells [6]. Currently, inhibitors of Hsp90 are thought to represent promising agents, providing a new class of drugs in cancer therapy. In parallel, recent studies indicate that the microenvironment of the tumour and activated fibroblasts in particular play a significant role in the process of tumourigenesis [7-9]. These cancer-associated fibroblasts (CAFs) may promote both tumour growth and progression [9,10]. In parallel, it has already been recognised that some oncological drugs may induce periodontium destruction, resulting in a permanent architectural defect [11,12]. Gingival fibroblasts represent the prevailing periodontal tissue cells while their injury during cancer therapy may determine pathology in periodontium. Nevertheless, data on effects of novobiocin on human fibroblasts still remain unavailable.

Taking the above into consideration, present investigations aimed at analysis of novobiocin effect on viability of human gingival fibroblasts (HGF-1).

## Materials and Methods

### Cell cultures

Gingival fibroblasts HGF-1 (CRL-2014, ATCC) were cultured in T-25 culture vessels (Nunc), in an incubators at the temperature of 37°C, in atmosphere of 5% CO_2_. Culture medium consisted of DMEM (ATCC) enriched with 10% FBS (Sigma-Aldrich).

### Fluorescence viability assay

Viability assays in gingival fibroblasts, HGF-1 employed the fluorescence test of Live⁄Dead Viability⁄Cytotoxicity Kit (Invitrogen, USA). The test allows to distinguish viable cells (stained with green-fluorescent calcein-AM) from dead cells (stained with red-fluorescent ethidium homodimer-1). In the studies novobiocin (Sigma-Aldrich) was used. The culture medium consisted of DMEM (ATCC) enriched with 10% FBS (Sigma-Aldrich). The studies took advantage of 24 h cultures of gingival fibroblasts, HGF-1, which following incubation were subjected to triple rinsing. The tests in triple repetitions were conducted in culture Lab-Tek Chamber Slides (Nunc) in presence of culture medium alone - control (0.5 × 10^6^ cells of HGF-1) and in presence of novobiocin (in concentrations of 0.1, 0.5, 1, 2.5 or 5 mM/L/0.5 × 10^6^ HGF-1 cells). The samples were incubated for 20 h at 37°C in presence of 5% CO_2_. In addition, the samples were incubated with 1 mM/L novobiocin for 5 and 10 h. Following the incubation the cells were rinsed with culture medium and their cell viability was assayed. The readout took advantage of the fluorescence microscope, Nikon Eclipse E200 (magnif. of 1000×).

### ATP assay

ATP content of HGF-1 gingival fibroblasts was evaluated using a luminescence test (CellTiter-Glo Luminescent Cell Viability Assay, Promega). The culture medium consisted of DMEM (ATCC), enriched with 10% FBS (Sigma-Aldrich). In the studies 24 h cultures of HGF-1 gingival fibroblasts were used, which following incubation were subjected to triple rinsing. The studies, in three repetitions, were conducted in culture medium alone – the control (10^5^ HGF-1 cells) and in presence of novobiocin (0.1, 0.5, 1, 2.5 or 5 mM/L/10^5^ HGF-1 cells). The prepared cells were incubated for 20 h at the temperature of 37°C in presence of 5% CO_2_. Subsequently, they were rinsed with culture medium and subjected to the test evaluating ATP content. The results were read out using a luminometer (GloMax, Promega). In presence of ATP a light is emitted which is read out in relative light units (RLU). Intensity of the emitted light quants is directly related to quantity of ATP present in the test. Viability of fibroblasts was calculated as a percentage of light intensity (RLU) emitted from experimental samples to intensity emitted from control samples, which was set as representing 100% viability.

### LDH release

The tests of LDH release were conducted using CytoTox 96 Non-Radioactive Cytotoxicity Assay kits (Promega, Madison). In the studies 24 h cultures of HGF-1 gingival fibroblasts were used which, following incubation were rinsed three times. The studies were performed in medium alone – the control (10^5^ HGF-1 cells) and in presence of novobiocin (0.1, 0.5, 1, 2.5 or 5 mM/L/10^5^ HGF-1 cells). The tubes were centrifuged at 500 rpm for 4 min at the temperature of 20°C. The prepared cells were incubated for 20 h at the temperature of 37°C in presence of 5% CO_2_. In addition the samples with novobiocin at the concentration of 1 mM/L were incubated for 5 and 10 hours. The studies were conducted as specified by the producer. The results were read out as absorbance at 492 nm. The percentage of cytotoxicity was calculated as a quotient of absorbance reflecting LDH release in experimental samples to absorbance value reflecting LDH release in samples with maximum lysis.

### Statistical methods

Results obtained in the studies were subjected to analysis using the STATISTICA 8 software for Windows operational system. Comparative analysis of ATP levels and studies on LDH release employed the unifactorial analysis of variance (one-way ANOVA) with the Tukey-Kramer’s test. Comparative analysis of gingival fibroblast viability took advantage of the non-parametric Mann-Whitney’s test and the Kruskal-Wallis’es test. A difference was considered significant when p < 0.05.

## Results

**Figure 1:**
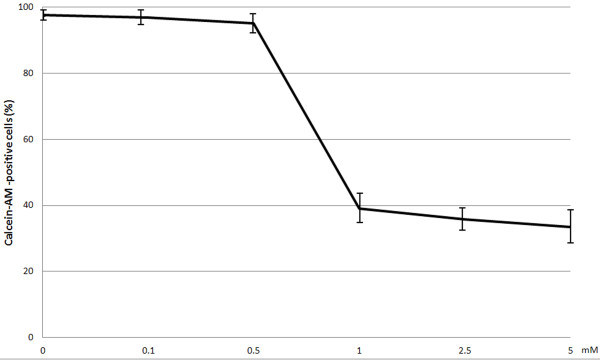
**Viability of gingival fibroblasts (fluorescence test) after 20 h incubation with examined novobiocin concentrations.** The studies were conducted in triple repetitions. The obtained results represent mean values ± SD (denoted in bars).

**Figure 2:**
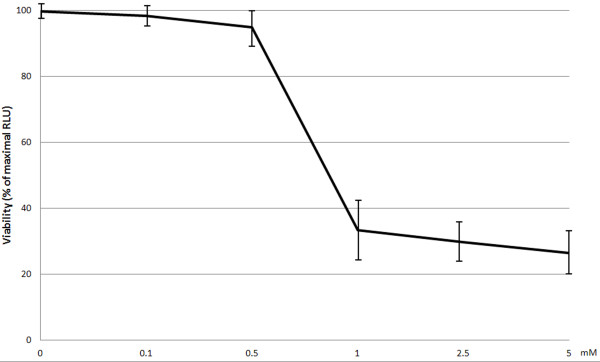
**Viability of gingival fibroblasts (ATP assay) after 20 h incubation with examined novobiocin concentrations.** The studies were conducted in triple repetitions. The obtained results represent mean values ± SD (denoted in bars).

In the studies percentage of viable HGF-1 fibroblasts in control samples using the fluorescence test ranged between 94 and 99% (97.6 ± 2.32%), while in ATP assay the average value in the control samples was taken as 100%. In neither of the tests a significant difference could be detected between viability in control samples and viability of fibroblasts incubated with novobiocin at concentrations of 0.1 mM or 0.5 mM. However, gingival fibroblast viability was markedly reduced (p < 0.0001) following application of novobiocin at the concentration of 1 mM. No significant differences in fibroblast viability were detected in samples treated with 1 mM, 2.5 mM or 5 mM novobiocin (Figures 1 and 2).

**Figure 3:**
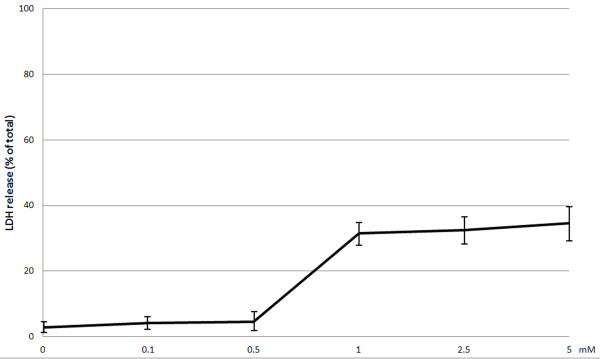
**Release of LDH in 20 h culture of gingival fibroblasts with the examined novobiocin concentrations.** The studies were conducted in triple repetitions. The obtained results represent mean values ± SD (denoted in bars).

In control samples the percentage of LDH-releasing cells ranged between 1 and 3.5% (2.8 ± 1.76%). No significant difference could be detected between LDH levels in the control samples and LDH levels in samples incubated with novobiocin at the concentrations of 0.1 mM or 0.5 mM. Levels of LDH were markedly increased (p < 0.0001) in fibroblast cultures subjected to novobiocin action at the concentration of 1 mM. No significant alterations were noted in LDH levels in samples incubated with 1 mM, 2.5 mM or 5 mM novobiocin (Figure 3).

**Figure 4:**
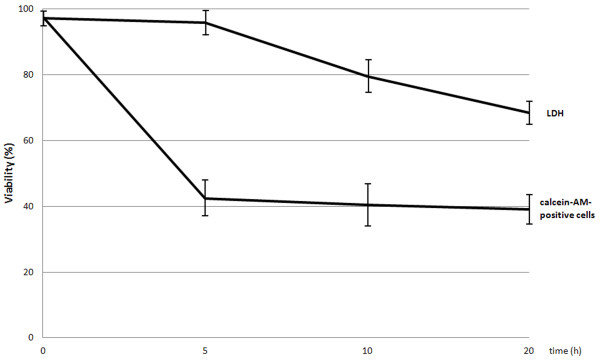
**Viability of gingival fibroblasts (fluorescence test and LDH test) in culture with 1 mM novobiocin after 5 h, 10 h and 20 h incubation.** The studies were conducted in triple repetitions. The obtained results represent mean values ± SD (denoted in bars).

Viability of HGF-1 fibroblasts treated with novobiocin at the concentration of 1 mM obtained in fluorescence test and that obtained in LDH release tests differed between each other: in fluorescence test viability of HGF-1 underwent a significant reduction already after 5 h and it did not change in consecutive time points of testing, in turn in LDH release assays the viability decreased significantly after 10 h culture and continued to increase till the 20th hour (Figure 4).

## Discussion

The Hsp90 plays essential roles in the folding, maturation and activity of many proteins that are involved in signal transduction and transcriptional regulation [5]. The proteins which are known to interact with Hsp90 include glucocorticoid receptors [13], Akt/Protein kinase B and Raf-1 [14], the tumor suppressor protein TP53 [15] and NOS family members [16]. The anti-cancer effects induced by novobiocin and its analogues through Hsp90 inhibition have already been well described [4,5]. Therefore, Hsp90 chemical inhibitors may find application in oncological therapy [17,18]. Moreover, application of Hsp90 inhibitors is considered in treatment of certain infectious diseases, because in eukaryotic cells Hsp90 is essential for the replication of obligatory intracellular parasites [19,20]. In recent years, development of neoplastic process has been found to be contributed by CAFs present in tumour stroma [9,10]. In parallel, fibroblasts represent prevailing connective tissue cells which provide integrity to its structure.

In this study we have examined for the first time effect of novobiocin on viability of human gingival fibroblasts (HGF-1), which are the dominant periodontal tissue cells [21,22]. Using a fluorescent test and ATP assay we have shown that novobiocin in doses of 0.1 and 0.5 mM failed to alter cell viability. Nevertheless, 0.5 mM novobiocin has insignificantly decreased viability of fibroblasts using ATP assay. The test represents the most sensitive assay of cell viability [23], which may explain differences in the obtained results. Using both tests we have demonstrated a significant decrease in HGF-1 viability following their 20 h incubation with 1 mM novobiocin. Percentage of viable fibroblasts following their 20 h incubation with 1.0, 2.5 and 5.0 mM novobiocin has amounted to 20-38% and it has not been dependent on the dose of novobiocin. In an earlier study by Calamia et al. [24], a significant reduction was noted in viability of human chondrocytes also in presence of 1 mM novobiocin. Results of Shelton et al. [25] contrast with those of ours. The authors demonstrated that novobiocin in concentration of 0.252 mM markedly inhibited proliferation of Jurkat T-lymphocytes, significantly reducing their viability. In turn, novobiocin at the dose of 0.4353 mM significantly reduced proliferation of K562 human erythroleukaemic cells [26]. However, the data pertain leukaemic cells, which as neoplastic cells may be particularly sensitive to Hsp90 inhibitors. The suggestion has been supported by reports showing that Hsp90 in cancer cells has a higher affinity for Hsp90 inhibition drugs than the Hsp90 in normal cells [27].

The demonstrated by us novobiocin-mediated decreased viability of fibroblasts seems to result from induction of an apoptotic response. The suggestion, in turn, corresponds with the data indicating that novobiocin and its derivatives may induce cell death along the apoptosis pathway [24,26,28]. In our study we have conducted also fibroblast viability tests following 5, 10 and 20 h treatment with 1 mM novobiocin, using for the purpose the fluorescence test and LDH assay. It has been already well established that stable cytoplasmic enzyme lactate dehydrogenase (LDH) is released from necrotic cells and, therefore, the use of LDH assay establishes cell death by necrosis [23]. After 5 h incubation of fibroblasts with 1 mM novobiocin, the percentage of viable cells in fluorescence test has been significantly reduced and has shown no statistical alterations in consecutive time points of testing. In turn, LDH release upon 1 mM novobiocin treatment has been significantly higher after 10 h than in corresponding control and increased further till 20 h. The results remain in contrast to the detected decrease in fibroblast viability already after 5 h exposure, using fluorescence test and ATP assay. This may indicate that LDH assay represents a test less sensitive than the remaining two tests applied in the study, in accordance to the earlier observation of Weyermann et al. [23]. However, on the other hand it also seems probable that novobiocin in concentration of at least 1 mM induces fibroblast apoptosis, leading after 10 hour incubation to post-apoptotic lysis of the fibroblasts. The phenomenon of post-apoptotic lysis has already been well described and referred to the post-apoptotic change as secondary necrosis [29,30].

## Conclusion

Data presented in this study indicate that novobiocin may induce death of human gingival fibroblasts. Therefore, application of this Hsp90 inhibitor drug in neoplastic therapy seems controversial: novobiocin on one hand reduces tumour-associated CAFs and, on the other, its application may lead to a severe destruction of periodontium.

## Competing interests

The authors declare no competing interests.

## Authors’ contributions

AKS and TMK: study conception and design, participation in performation of the experimental studies and data analysis, drafting of the manuscript; AS: coordination and help in interpretation of the study results. All authors read and approved the final manuscript.

## Pre-publication history

The pre-publication history for this paper can be accessed here:

http://www.biomedcentral.com/2050-6511/15/25/prepub
